# Variation in gene duplicates with low synonymous divergence in *Saccharomyces cerevisiae *relative to *Caenorhabditis elegans*

**DOI:** 10.1186/gb-2009-10-7-r75

**Published:** 2009-07-13

**Authors:** Vaishali Katju, James C Farslow, Ulfar Bergthorsson

**Affiliations:** 1 Department of Biology, Castetter Hall, 1 University of New Mexico, Albuquerque, NM 87131-0001, USA

## Abstract

**Background:**

The direct examination of large, unbiased samples of young gene duplicates in their early stages of evolution is crucial to understanding the origin, divergence and preservation of new genes. Furthermore, comparative analysis of multiple genomes is necessary to determine whether patterns of gene duplication can be generalized across diverse lineages or are species-specific. Here we present results from an analysis comprising 68 duplication events in the *Saccharomyces cerevisiae *genome. We partition the yeast duplicates into ohnologs (generated by a whole-genome duplication) and non-ohnologs (from small-scale duplication events) to determine whether their disparate origins commit them to divergent evolutionary trajectories and genomic attributes.

**Results:**

We conclude that, for the most part, ohnologs tend to appear remarkably similar to non-ohnologs in their structural attributes (specifically the relative composition frequencies of complete, partial and chimeric duplicates), the discernible length of the duplicated region (duplication span) as well as genomic location. Furthermore, we find notable differences in the features of *S. cerevisiae *gene duplicates relative to those of another eukaryote, *Caenorhabditis elegans*, with respect to chromosomal location, extent of duplication and the relative frequencies of complete, partial and chimeric duplications.

**Conclusions:**

We conclude that the variation between yeast and worm duplicates can be attributed to differing mechanisms of duplication in conjunction with the varying efficacy of natural selection in these two genomes as dictated by their disparate effective population sizes.

## Abstract 2

Differences between yeast and worm duplicates result from differences in mechanisms of duplication and effective population size.

## Background

Gene duplication is widely regarded as one of the major contributing factors to the origin of novel biochemical processes and new lineages bearing morphological innovations during the course of evolution [1-10]. The direct examination of large, unbiased samples of young gene duplicates in the early stages of evolution is crucial to understanding the origin, preservation and diversification of new genes. The phylogenetic breadth of completed sequencing projects is now sufficient to enable comparisons of gene duplication patterns across diverse taxa and determine whether the structural/genomic features of gene paralogs are lineage-specific or display phylogenetic independence. Additionally, if gene duplicate patterns and features do vary markedly amongst diverse taxa, it begs the question as to which evolutionary forces are paramount in driving this inter-taxa variation.

In preceding studies, one of us investigated the structural features and other genomic attributes of a large sample of evolutionarily young gene duplicates in the nematode *Caenorhabditis elegans *in an attempt to further infer the dominant patterns of gene duplication within this genome [11,12]. Despite observable diversity among gene duplicate pairs with regard to the structural and genomic features under scrutiny, some dominant patterns were apparent. First, newly originated gene duplicates tend to arise intra-chromosomally relative to the progenitor copy, often present in tandem placement. Second, aside from a few segmental-scale duplications, gene duplication tracts tended to be relatively compact, often failing to encompass open reading frames (ORFs) in their entirety and resulting in the creation of structurally heterogeneous gene duplicates relative to the progenitor locus. Third, structural heterogeneity between paralogs, manifested as one or both paralogs containing unique exonic regions to the exclusion of the other copy, was evident even in the newborn cohort of gene duplicates despite zero synonymous divergence over their homologous regions. Fourth, newborn duplicates were often observed as adjacent loci in inverted orientation, suggesting that inversions may be part and parcel of the original duplication event. As a first step towards determining whether these patterns of gene duplication are prevalent in other eukaryotic genomes, we conducted a similar analysis of gene duplicates with low synonymous divergence in the genome of the budding yeast, *Saccharomyces cerevisiae*.

The evolution of redundant sequences in the *S. cerevisiae *genome differs in several notable ways from their counterparts in *C. elegans*. Most importantly, the yeast genome has multiple duplicated segments that are remnants of a single ancestral whole-genome duplication (WGD) event preceding the divergence of the *Saccharomyces sensu stricto *species complex with subsequent genome-wide deletions resulting in the restoration of functional normal ploidy [13-21]. It is important to recognize that the cohort of gene duplicate pairs with low synonymous divergence in the *S. cerevisiae *genome comprises a mixed population of evolutionarily older gene duplicates homogenized by the action of codon usage bias selection and/or gene conversion, and gene duplicates of possibly recent evolutionary origins. Hence, where possible, we conduct analyses at three levels: the cumulative dataset comprising both evolutionarily older and recently derived gene duplicate pairs; putative evolutionarily older gene duplicates residing within duplicated blocks referred to as 'ohnologs' as per Wolfe [22,23] (we follow that nomenclature here); and putative evolutionarily recent gene duplicates (henceforth referred to as 'non-ohnologs'). Preceding studies have referred to ohnologs and non-ohnologs as WGD and small-scale duplication (SSD) genes, respectively [24-26].

## Results

### Final data set

The final data set considered in this study is composed of 68 duplication tracts comprising 93 duplicate pairs with K_*S *_values ranging from 0 to 0.35 (Tables 1 and 2). Of these 68 cases, 56 appear to constitute single-locus gene duplications (Table 1). The other 12 duplication events comprise what we classify as 'linked sets' involving the duplication of more than one gene locus (Table 2). The duplication of these 12 linked sets resulted in an additional 37 gene duplicate pairs (minimum estimate).

**Table 1:** List of 56 gene duplications in *S. cerevisiae *with K_*S *_< 0.35 that appear to span a single locus only

Duplicate pair	K_*S*_	Structural category	Chromosomal location	Duplication span (bp)	5' homology (bp)	3' homology (bp)
*YPL220W/YGL135W	0.0000	Complete	XVI/VII	657	3	0
*YBR031W/YDR012W	0.0038	Complete	II/IV	1,102	1	12
*YDR342C/YDR343C	0.0052	Complete	IV/IV	1,896	97	86
*YPR080W/YBR118W	0.0066	Complete	XVI/II	1,381	0	4
*YOR133W/YDR385W	0.0072	Complete	XV/IV	2,533	0	3
YMR321C/YPL273W	0.0155	Chimeric	XIII/XVI	510	0	197
*YDL182W/YDL131W	0.0222	Chimeric	IV/IV	1,220	0	0
*YJL138C/YKR059W/	0.0237	Complete	X/XI	1,192	4	0
YIL177C/YLR462W_464W_466W^†^	0.0238	Complete	IX/XII	6,907	816	423
*YBR181C/YPL090C	0.0388	Complete	II/XVI	1,107	4	0
*YDL136W/YDL191W	0.0395	Complete	IV/IV	858	2	2
YBL107W-A/YER138W-A	0.0435	Complete	II/V	310	153	52
*YNL209W/YDL229W	0.0612	Complete	XIV/IV	1,883	6	35
*YDL184C/YDL133C-A/	0.0612	Complete	IV/IV	113	19	17
*YBL072C/YER102W	0.0817	Complete	II/V	607	3	1
*YJR145C/YHR203C	0.0854	Complete	X/VIII	1,060	6	1
*YHR141C/YNL162W	0.0918	Complete	VIII/XIV	843	10	0
*YPR156C/YGR138C	0.0985	Chimeric	XVI/VII	1,419	0	0
YNL030W/YBR0009C	0.1062	Complete	XIV/II	353	41	0
*YJR009C/YGR192C	0.1123	Complete	X/VII	1,055	56	0
YAL005C/YLL024C	0.1147	Complete	I/XII	1,931	0	2
*YGL076C/YPL198W	0.1196	Complete	VII/XV1	1,658	5	0
*YPR102C/YGR085C	0.1237	Complete	XVI/VII	534	6	3
*YER074W/YIL069C	0.1333	Complete	V/IX	876	2	1
*YHL001W/YKL006W	0.1429	Complete	VIII/XI	819	1	2
*YIL018W/YFR031C-A	0.1523	Complete	IX/VI	1,167	2	0
*YGR118W/YPR132W	0.1546	Complete	VII/XVI	810	4	3
*YDL131W/YDL182W	0.1768	Chimeric	IV/IV	1,232	0	0
*YLL045C/YHL033C	0.1809	Complete	XII/VIII	774	0	3
*YDR447C/YML024W	0.1896	Complete	IV/XIII	812	3	0
YGL258W/YOR387C	0.1939	Complete	VII/XV	1,424	796	7
*YNL301C/YOL120C	0.1955	Complete	XIV/XV	1,009	1	0
*YBR048W/YDR025W	0.1987	Complete	II/IV	985	3	0
*YLR287C-A/YOR182C	0.2022	Complete	XII/XV	628	5	0
*YNL302C/YOL121C	0.2076	Complete	XIV/XV	991	5	0
YIL029C/YPR071W	0.2154	Chimeric	IX/XVI	659	149	0
YGL147C/YNL067W	0.2490	Complete	VII/XIV	576	0	0
*YDR450W/YML026C	0.2491	Complete	IV/XIII	881	4	1
*YMR242C/YOR312C	0.2504	Complete	XIII/XV	1,001	5	0
*YBR191W/YPL079W	0.2508	Complete	II/XVI	910	5	1
*YBL027W/YBR084-C	0.2698	Complete	II/II	1,079	3	0
*YDR312W/YHR066W	0.2703	Complete	IV/VIII	1,362	0	0
*YDL083C/YMR143W	0.2838	Complete	IV/XIII	984	4	4
*YEL034W/YJR047C	0.2838	Complete	V/X	475	0	1
*YGR034W/YLR344W	0.2841	Complete	VII/XII	862	0	1
*YGL031C/YGR148C	0.2862	Complete	VII/VII	471	0	3
*YDL082W/YMR142C	0.2970	Complete	IV/XIII	1,007	3	2
*YLR448W/YML073C	0.2992	Complete	XII/XIII	958	10	2
*YLR029C/YMR121C	0.3061	Complete	XII/XIII	619	4	0
*YMR230W/YOR293W	0.3132	Complete	XIII/XV	771	15	1
*YLR441C/YML063W	0.3170	Complete	XII/XIII	774	5	1
*YCR024C-A/YEL017C-A	0.3176	Complete	III/V	137	5	0
*YGR027C/YLR333C	0.3187	Complete	VII/XII	345	17	1
YHR043C/YHR044C	0.3245	Complete	VIII/VIII	776	1	34
*YMR186W/YPL240C	0.3319	Complete	XIII/XVI	2,132	2	0
YDL075W/YLR406C	0.3363	Complete	IV/XII	768	3	2

Columns 1 and 2 list the systematic names of the two paralogs in question as per the *Saccharomyces *Genome Database. *A gene duplicate pair that has been classified as an ohnolog resulting from a WGD event. ^†^An ancestrally single locus that currently exists as three adjacent genes due to frame-shift mutations. Column 3 lists the synonymous-site divergence (K_*S*_) between the two paralogs as computed by the Nei and Gojobori method with a correction for multiple hits. Column 4 lists the particular category of structural resemblance (complete, partial or chimeric). Column 5 lists the chromosomal location of paralogs 1 and 2, respectively. Column 6 provides a minimal estimate of the length of the duplicated region, based on current visual inspection of the extent of sequence homology across the paralogs' coding and flanking regions. Columns 7 and 8 list the extent of discernible sequence homology between the paralogs in their 5' and 3' flanking regions, respectively.

**Table 2:** List of 12 linked sets involving the duplication of more than one gene locus in *S. cerevisiae *with K_*S *_< 0.35

Linked set	Paralogous set A	Paralogous set B	K_*S*_	Average K_*S*_	Structural categories	Chromosomal location	Duplication span (bp)
1	YLR154C-H	YLR157C-C	0.0000	0.0000	Complete	XII/XII	7,167
	YLR155C	YLR158C	0.0000		Complete		
	YLR156W	YLR159W	0.0000		Complete		
	YLR156C-A	YLR159C-A	0.0000		Complete		
	YLR157C	YLR160C	0.0000		Complete		
	YLR157W-D	YLR161W	0.0000		Partial		
							
2	YHR053C	YHR055C	0.0000	0.0000	Complete	VIII/VIII	1,816
	YHR054C	YHR056C	0.0000		Partial		
							
3	YCL065W	YCR041W	-	0.0019	Chimeric	III/III	2,509
	YCL066W	YCR040W	0.0000		Complete		
	YCL067C	YCR039C	0.0000		Complete		
	YCL068C	YCR038C	0.0058		Chimeric		
							
4	YNL033W	YNL019C	0.0000	0.0077	Complete	XIV/XIV	4,247
	YNL034W	YNL018C	0.0450		Complete		
							
5	YAR073W/75W	YHR216W	0.1074	0.0087	Complete	I/VIII	7,445
	YAR071W	YHR215W	0.0069		Complete		
	YAR070C	YHR214C-B	0.0000		Complete		
	YAR069C	YHR214C-D	0.0000		Complete		
							
6*	YKR106W	YCL073C	0.0359	0.0243	Complete	XI/III	6,928
	YKR105C	YCL069W	0.0127		Complete		
							
7	YDR543C	YER188C	0.0608	0.0377	Complete	IV/V	7,481
	YDR545W	YER189W	0.0147		Complete		
							
8	YJR162C	YNL337W	0.0000	0.0409	Complete	X/IV	2,916
	YJR161C	YNL336W	0.0818				
							
9	YCR107W/AAD3	YOL165C	0.0430	0.0430	Complete	III/XV	5,952
	YCR108C	YOL166W	-		Complete		
							
10	YAR050W	YHR211W	0.3081	0.0449	Complete	I/VIII	19,614
	YAR060C	YHR212C	0.0000		Complete		
	YAR061W	YHR212W-A	0.0000		Complete		
	YAR062W	YHR213W	0.0000		Complete		
	YAR064W	YHR213W-B	0.0000		Complete		
	YAR066W	YHR214W	0.0066		Complete		
	YAR068W	YHR214W-A	0.0000		Complete		
							
11	YNR073C	YEL070W	0.0482	0.0817	Complete	XIVI/V	4,611
	YNR072C	YEL069C	0.1152		Complete		
							
12	YAR033W	YGL051W	0.0280	0.0973	Complete	I/VII	6,461
	YAR031W	YGL053W	0.1667		Complete		

Columns 2 and 3 list the systematic names of the group of loci representing each paralogous set as per the *Saccharomyces *Genome Database. Column 4 lists the synonymous-site divergence (K_*S*_) between two paralogs within a linked set as computed by the Nei and Gojobori method with a correction for multiple hits. Column 5 presents the averaged K_*S *_value for all paralogous pairs within a linked set. Column 6 lists the particular category of structural resemblance (complete, partial or chimeric) for each duplicate pair. Column 7 lists the chromosomal location of paralogs 1 and 2, respectively. Column 8 provides a minimal estimate of the length of the duplicated region, based on current visual inspection of the extent of sequence homology across the paralogs' coding and flanking regions. *A linked set that has been classified as an ohnolog resulting from a WGD event. Dashes indicate an inability to compute synonymous divergence between the paralogs due to an altered reading frame in one or both gene copies.

Of the 56 single-locus gene duplication events, all but 10 have been previously characterized as paralogous *S. cerevisiae *gene pairs or ohnologs resulting from a WGD event [17-19,23]. In contrast, 11 of the 12 linked sets are thought to have originated from more localized, SSD events, as is the case for 10 single-locus duplication events. We seek to make the distinction between putative ohnologs and non-ohnologs in order to investigate if the genomic and structural features of these two classes of gene duplicates in the *S. cerevisiae *genome differ significantly.

### The majority of duplication events appear to span a single locus

**Figure 1:**
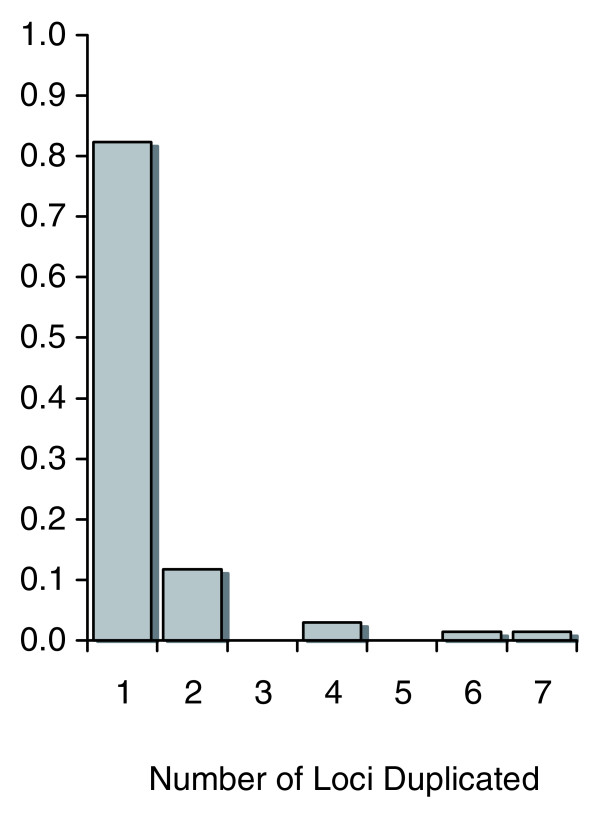
Frequency distribution of the minimum number of loci duplicated. The data set comprises 68 duplication events in the *S. cerevisiae *genome. The displayed data encompass ohnologs and non-ohnologs, duplications of a single-locus as well as multiple loci in the same duplication events (linked sets).

The determination of the extent of sequence homology between paralogs in their 5' and 3' flanking regions enabled us to determine a minimum estimate for the number of loci duplicated in a given duplication event. The range for the minimum number of loci duplicated is one to seven genes. In most cases, the duplication event appeared to span only a single locus (Figure 1). Together, duplication events leading to linked sets (duplication of two or more genes in one event) comprised 18% of all duplication events.

We bring these patterns to attention with the caveat that the extent of sequence homology discernible between two paralogs may not reflect the ancestral duplication span. This is particularly salient given that some *S. cerevisiae *paralogs thought to be evolutionarily older appear to be of recent origin (low levels of synonymous sequence divergence) due to the homogenizing effects of gene conversion and/or codon usage bias [19,27,28]. In these cases, while the original duplication event may have encompassed large segments of DNA or entire chromosomes (as would be the case for ohnologs), subsequent sequence divergence at selectively neutral sites, intergenic deletions as well as local rearrangements over evolutionary time will serve to diminish the extent of discernible sequence homology between the two copies, particularly in flanking regions, thereby leading to an underestimation of the number of loci encompassed in the ancestral duplication event.

Interestingly, all but one of the twelve linked sets involving the duplication of multiple loci are considered non-ohnologs (Table 2). If these duplication events have occurred subsequent to the WGD event within the *S. cerevisiae *lineage, their presence suggests that duplication events spanning multiple loci are relatively frequent and/or selectively advantageous within this genome. In contrast, 46 of the 56 single-locus duplications have been previously classified as ohnologs, indicating an erosion of sequence homology between the two paralogs in their intergenic regions in the post-duplication period.

### Most *S. cerevisiae *paralogs reside on different chromosomes

**Figure 2:**
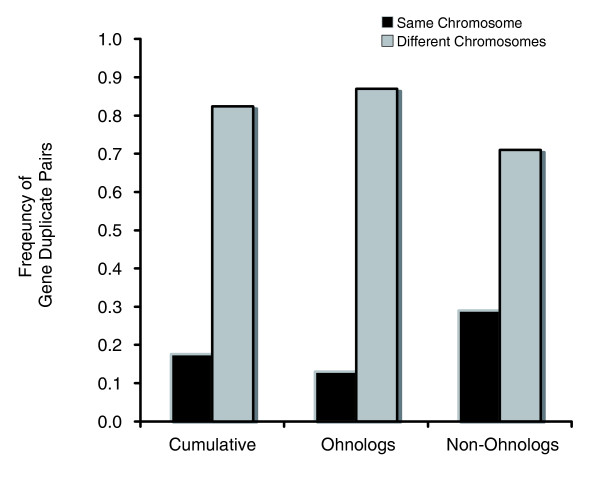
Frequencies of *S. cerevisiae *gene-duplicate pairs with both paralogs residing on the same chromosome versus different chromosomes. Results are displayed for the cumulative data (ohnologs and non-ohnologs), ohnologs only and non-ohnologs only.

With respect to genomic location, we determined whether the two paralogs comprising a gene duplicate pair reside on the same chromosome versus different chromosomes (Figure 2) for the cumulative data, ohnologs in isolation and non-ohnologs in isolation. Within the cumulative data set comprising both ohnologs and non-ohnologs (n = 68 duplication events), the two paralogs reside on different chromosomes in the majority of cases (82%; 56 of 68 duplicate pairs).

A comparison of ohnologs versus non-ohnologs in isolation with respect to the chromosomal location of paralogs appears to yield differential frequencies of paralogs on the same versus different chromosomes between these two classes of gene duplicates. Eighty-seven percent of all ohnologs comprise paralogs residing on different chromosomes. The remaining 13% of ohnologs comprising paralogs located on the same chromosome must be owing to secondary movement in the post-duplication period, if these duplicate pairs did indeed originate from a WGD event or whole-chromosomal duplications. Non-ohnologs appear to comprise fewer gene duplicate pairs, with paralogs residing on different chromosomes (71%) relative to ohnologs. However, a *G*-test for goodness of fit revealed no significant differences in the chromosomal location of ohnologs versus non-ohnologs (*G*_*adj *_= 2.18, *d.f*. = 1, 0.1 <*P *< 0.5). Hence, we cannot reject the null hypothesis that the chromosomal location of paralogs (same versus different chromosomes) is independent of whether they arose from the WGD event or not, with extant *S. cerevisiae *paralogs more likely to exist on different chromosomes.

### Preponderance of complete duplicates

**Figure 3:**
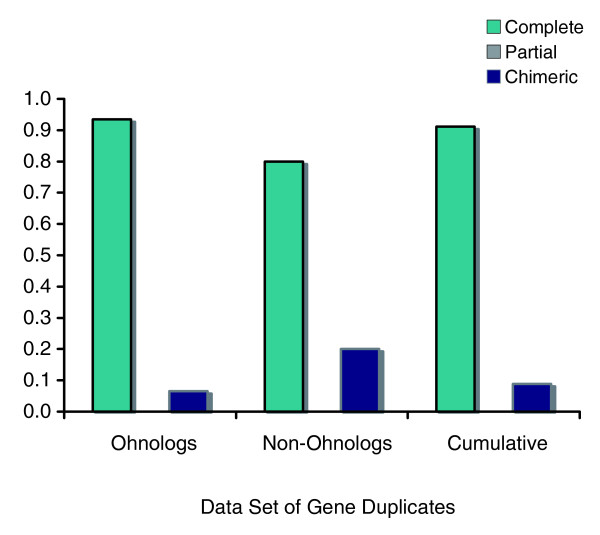
Composition frequencies of three structural categories of gene duplicates within the *S. cerevisiae *genome. Results are displayed for ohnologs only, non-ohnologs only and the cumulative data (ohnologs and non-ohnologs). Methodology for the structural characterization of gene duplicates is based on [11].

A direct comparison of the intron/exon structure of the paralogs across the 56 single-locus duplication events comprising both ohnologs and non-ohnologs revealed most gene duplicates in this data set (91%) as complete duplicates, with an absolute absence of partial duplicates and a low incidence of duplicates with chimeric structure (Figure 3). Among the 47 ohnologs, only two pairs exhibit structural heterogeneity (both chimeric). The frequency of structurally heterogeneous duplicate pairs within the non-ohnologs class thought to have originated from SSD events is slightly different. Of these 21 non-ohnologs, 10 (48%) and 11 (52%) comprise what appear to be single-locus duplications and linked sets, respectively. Only one of the ten putative single-locus duplication events involving non-ohnologs exhibits a chimeric structure. Of the 11 linked sets, eight comprise complete duplications of all loci duplicated within that particular duplication event (range of number of loci duplicated is two to seven). The remaining three linked sets are characterized as: two linked sets (of two and six simultaneously duplicated loci, respectively) wherein one terminal/flanking locus within the duplication tract displays a partial structure; and one linked set of four loci wherein both terminal/flanking loci exhibit a chimeric structure. Cumulatively speaking, only 18% (4 of 22) of non-ohnologs in yeast display some facet of structural heterogeneity. Moreover, there is no significant difference in the frequencies of these three structural categories when the data set is further partitioned on the basis of ohnologs versus non-ohnologs (*G*_*adj *_= 1.26, *d.f*. = 1, 0.1 <*P *< 0.5).

### Reduced duplication span in ohnologs relative to non-ohnologs

**Figure 4:**
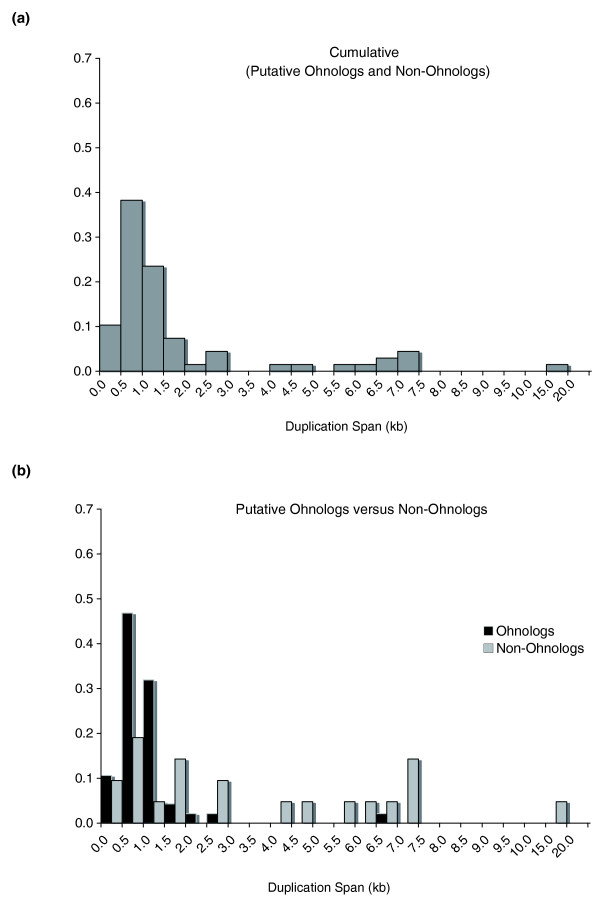
Distribution of minimum duplication spans (in kilobases) for *S. cerevisiae *gene-duplicate pairs with synonymous-site divergence of 0 ≤ K_*S *_< 0.35. **(a) **Cumulative data set comprising both ohnologs and non-ohnologs (n = 68 duplication events). **(b) **Data set partitioned into ohnologs (n = 47 duplication events) and non-ohnologs (n = 21 duplication events).

Figure 4a illustrates the distribution of duplication spans for all 68 duplications events. The range of duplication spans for the composite data set (n = 68) is 113 to 19,614 bp with a median value of 1,004 bp. All but one of the duplication span values were < 7.5 kb, with the lone exception spanning approximately 19.6 kb. The L-shaped distribution implies that the discernible extent of duplication is relatively short for extant yeast duplicates and this pattern could be due to the duplication of relatively short sequence tracts and/or the duplication of lengthier sequence tracts with subsequent erosion of sequence homology in the flanking regions of paralogs over evolutionary time (due to sequence divergence or intergenic deletions), as would be the case for paralogs resulting from the ancient WGD event or segmental duplication events.

We investigated whether ohnologs and non-ohnologs differ significantly with respect to their duplication spans (Figure 4b). For instance, one might expect that gene duplicates owing their origin to the WGD event, on average, tend to have lengthier duplication spans relative to non-ohnologs. The frequency distribution of extant duplication spans for ohnologs appears to be restricted to short sequence tracts ranging from 113 bp to 6.9 kb with a median value of 984 bp. Approximately 66% of all duplication span values for ohnologs fall short of the median gene length of 1,071 bp in *S. cerevisiae*. In contrast, the duplication spans of non-ohnologs are dispersed across a wider range of values (310 bp to 19.6 kb) with a median value of approximately 2.5 kb, which greatly exceeds the median gene length in *S. cerevisiae*. In addition, the duplication spans of ohnologs and non-ohnologs were found to differ significantly (Wilcoxon two-sample test, *P *= 0.0003).

### Limited sequence homology in flanking regions

The nucleotide sequences of 5' and 3' flanking regions for each of the two paralogs within each duplicate pair were aligned to determine the duplication termination points. This also enabled the determination of the extent of sequence homology between the paralogs in their upstream and downstream flanking regions. The extent of 5' and 3' flanking region homology between paralogs was calculated for 56 duplicate pairs that appear as single-locus duplications. The 12 linked sets comprising the simultaneous duplication of multiple genes were excluded from this analysis.

**Figure 5:**
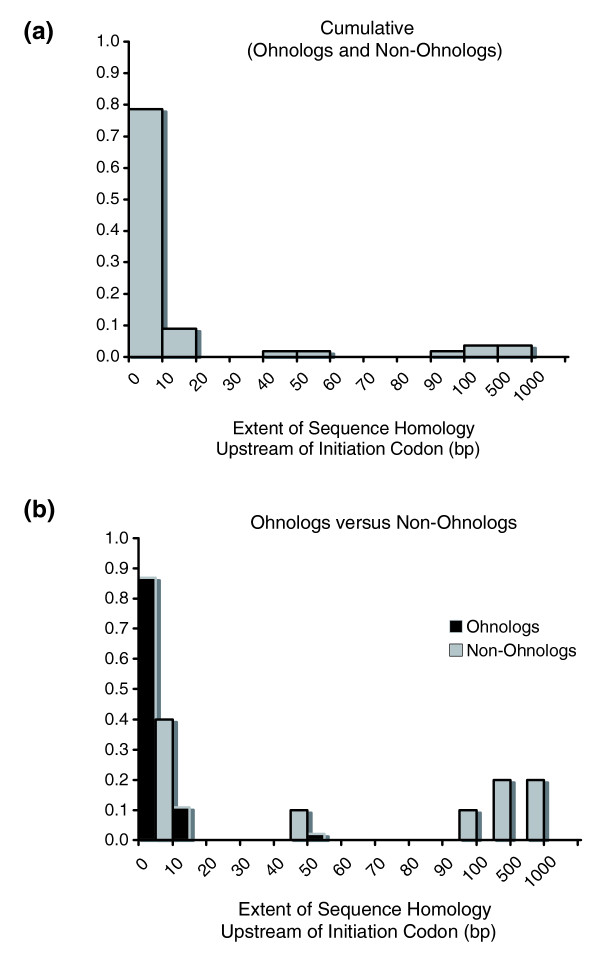
Distribution of the extent of discernible sequence homology between paralogs (in base pairs) upstream of the initiation codon. Gene duplicates comprising the 12 linked sets were excluded in this analysis. **(a) **Cumulative data set comprising both ohnologs and non-ohnologs (n = 56 duplication events). **(b) **Data set partitioned into ohnologs (n = 46 duplication events) and non-ohnologs (n = 10 duplication events).

The frequency distribution of the extent of 5' sequence homology between two paralogs for n = 56 duplicate pairs is displayed in Figure 5a. For approximately 80% of duplicate pairs, the detectable sequence homology in the 5' region is limited to 0 to 10 bp. The range of discernible 5' sequence homology between paralogs in this data set is 0 to 816 bp with a median value of 3.5 bp. A comparison of the very same distributions for putative ohnologs versus non-ohnologs (Figure 5b) demonstrates that, on average, both these classes of duplicate pairs exhibit a similar L-shaped distribution of extremely limited 5' sequence homology between paralogs, with a range of 0 to 56 bp and 0 to 816 bp, respectively. Although the 5' sequence homology distribution for ohnologs appears to have a far greater right skew relative to that for non-ohnologs, these two classes of gene duplicates were not found to be statistically different with respect to the extent of 5' sequence homology between paralogs (Wilcoxon two-sample test, *P *= 0.1253).

**Figure 6:**
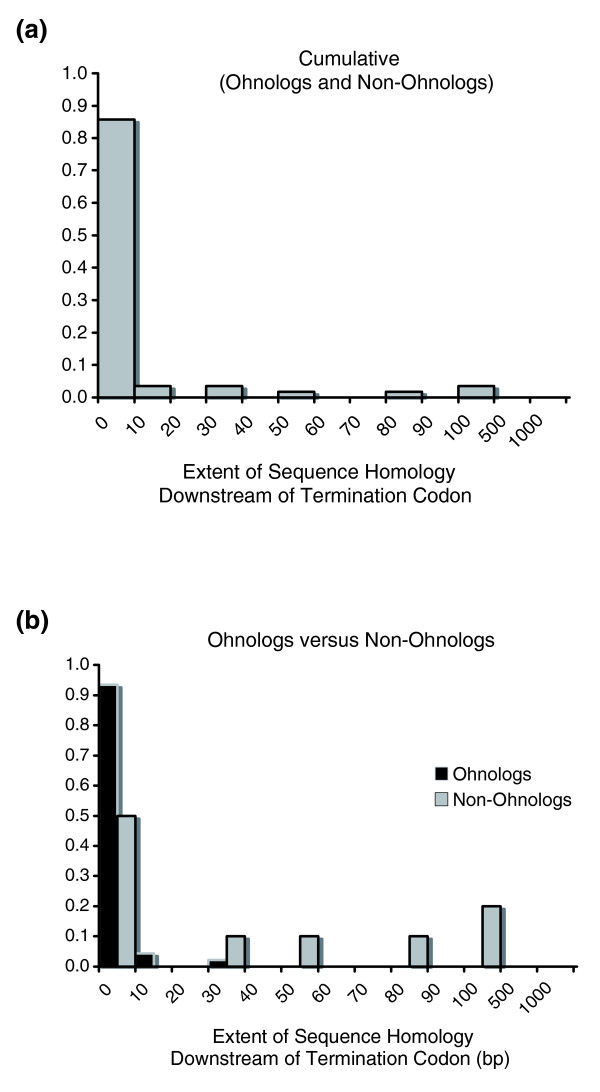
Distribution of the extent of discernible sequence homology between paralogs (in base pairs) downstream of the termination codon. Gene duplicates comprising the 12 linked sets were excluded in this analysis. **(a) **Cumulative data set comprising both ohnologs and non-ohnologs (n = 56 duplication events). **(b) **Data set partitioned into ohnologs (n = 46 duplication events) and non-ohnologs (n = 10 duplication events).

The distribution of extant 3' sequence homology between paralogs comprising the 56 single-locus duplication events mirrors that observed for 5' flanking regions (Figure 6), if not more downwardly biased. Approximately 86% of duplicate pairs have detectable 3' sequence homology limited to a mere 0 to 10 bp. The range of discernible 3' sequence homology between paralogs in this data set is 0 to 423 bp with a median value of a mere 1 bp. When the data are further differentiated into ohnologs and non-ohnologs, these two classes of duplicate pairs are found to differ significantly with respect to the extent of 3' sequence homology between paralogs (Wilcoxon two-sample test, *P *= 0.0172). *Ohnologs *appear to have more restricted 3' sequence homology relative to non-ohnologs with a median value of 1 bp and a range of 0 to 35 bp. In contrast, the median value and range of 3' sequence homology for non-ohnologs is 20.5 bp and 0 to 423 bp, respectively. Taken together, *S. cerevisiae *paralogs exhibit extremely limited tracts of sequence identity in their 5' and 3' flanking regions.

### Intron preservation in paralogs

Intron-bearing genes comprise only 4% of the total ORFs found in the *S. cerevisiae *genome [29]. In contrast, our data set of gene duplicates contains an unusually high frequency of genes with introns (25 of 93; approximately 27%). These intron-containing genes are overwhelmingly ribosomal proteins, which, in turn, comprise a significant fraction of this data set.

We found no cases of intron loss in the gene duplicates analyzed here. Half of the ohnologs (22 of 44 cases) appearing as single-locus duplications contain intron(s) that have been retained in both copies. Three pairs of non-ohnologs comprising a single-locus duplication also contain introns. In each of these three cases, the two copies reside on different chromosomes. Therefore, we do not have any evidence that retrotransposition contributes to duplicates that occur in radically different locations in the yeast genome.

### The incidence of highly diverged introns in ribosomal protein duplicates

Our sequence alignments of paralogs across their flanking regions, exons and introns revealed an interesting observation, namely the presence of nonhomologous introns between paralogs across 24 pairs of ribosomal protein duplicates with varying *K*_*S *_values (ranging from approximately 0.039 to 0.336) that have all previously been characterized as ohnologs (Table 3). These represent 35% of the duplication events in this dataset. In each case, the exonic regions are conserved in addition to short tracts of the intron(s) near the splice junctions. Most of the intronic regions appear nonhomologous between the two paralogs and are characterized by both nucleotide sequence and size differences. It is possible that this divergence in intronic sequences represents some form of intron conversion event. Alternatively, a more plausible scenario is that the paralogs are evolutionarily older than they appear based on their *K*_*S *_values with a saturation of substitutions in the intronic regions that are presumably under no selection for sequence conservation. The conservation of short intronic sequence tracts between the paralogs in the vicinity of their splice junctions suggests strong purifying selection for the maintenance of correct sequence signals for the accurate excision of introns by the RNA splicing machinery.

**Table 3:** Summary of 24 *S. cerevisiae *ribosomal protein paralogs with largely nonhomologous intronic sequences despite relatively low levels of synonymous divergence

					I1 (bp)				I2 (bp)			
								
Duplicate pair	K_*S*_	5' homology (bp)	3' homology (bp)	E1 (bp)	5' H	NH	3' H	E2 (bp)	5' H	NH	3' H	E3 (bp)
YDL075W/YLR406C	0.3363	3	2	57	6	415/343	0	285		-		-
YMR230W/YOR293W	0.3132	15	1	52	8	400/427	2	266		-		-
YLR448W/YML073C	0.2992	10	2	15	6	375/406	3	516		-		-
YDL082W/YMR142C	0.2970	3	2	8	2	358/395	5	592		-		-
YGR034W/YLR344W	0.2841	0	1	19	6	68/438	3	365		-		-
YDL083C/YMR143W	0.2838	4	4	24	7	423/535	2	408		-		-
YBL027W/YBR084C	0.2698	3	0	2	6	370/492	8	568		-		-
YBR191W/YPL079W	0.2508	5	1	11	7	377/410	4	472		-		-
YMR242C/YOR312C	0.2504	5	0	1	2	467/416	8	518		-		-
YDR450W/YML026C	0.2491	4	1	47	8	424/390	3	394		-		-
YNL302C/YOL121C	0.2076	5	0	20	10	539/378	2	415		-		-
YLR287C-A/YOR182C	0.2022	5	0	3	6	420/401	4	189		-		-
YBR048W/YDR025W	0.1987	3	0	45	7	502/330	2	426		-		-
YNL301C/YOL120C	0.1955	1	0	112	6	424/439	2	449		-		-
YDR447C/YML024W	0.1896	3	0	3	6	298/382	10	408		-		-
YGR118W/YPR132W	0.1546	4	3	65	6	312/357	2	373		-		-
YIL018W/YFR031C-A	0.1523	2	0	4	6	391/138	3	761		-		-
YIL001W/YKL006W	0.1429	1	2	129	7	318/318	74	288		-		-
YER074W/YIL069C	0.1333	2	1	3	6	458/401	2	405		-		-
YGL076C/YPL198W	0.1196	5	0	11	6	451/400	12	94	7	456/395	5	630
YHR141C/YNL162W	0.0918	10	0	4	5	433/504	2	317		-		-
YJR145C/YHR203C	0.0854	6	1	14	7	247/260	2	772		-		-
YDL136W/YDL191W	0.0395	2	2	3	4	387/473	12	360		-		-
YBR181C/YPL090C	0.0388	4	0	6	4	336/378	10	705		-		-

Column 3 and 4 list the length of the extent of discernible homology between the two paralogs upstream of the initiation codon and downstream of the termination codon, respectively. Columns 5, 9 and 13 (E1, E2 and E3) list the length of exons 1, 2 and 3 (where applicable), respectively. Columns 6 to 8 provide details about the extent of homology between the two paralogs across intron 1. Columns 6 and 8 list the length of the short tracts of homology in the 5' and 3' ends of intron 1 near the splice junctions. Column 7 lists the length of the nonhomologous tracts of intron 1 for both paralogs. Columns 10 to 12 list similar details for intron 2, where present.

## Discussion

Given the importance of gene duplication to the origin of biological innovations, a deeper understanding of the evolutionary process might be gained from investigating the differential contributions, if any, of gene duplication to the genome architecture within diverse lineages. Genomes can be variably shaped by the mutational input of duplicate sequences (the frequency and the flavor of redundant genetic sequences being generated) and their differential preservation/degeneration dictated by the strength of natural selection and random genetic drift. Some effort has been made towards such comparative genomic analyses of the gene duplication process, both at the level of closely and distantly related eukaryotic genomes (for example, [30-42]). In a similar vein, this study analyzes various structural and genomic features of gene duplicates in the *S. cerevisiae *genome and aims to contrast these with gene duplicates with low synonymous divergence in the genome of a multicellular eukaryote, *C. elegans*, as well as compare evolutionarily recent gene duplications with evolutionarily older gene duplicates with low synonymous divergence in *S. cerevisiae*.

Most of the *S. cerevisiae *duplication events (approximately 69%; 47 of 68) analyzed here are thought to have originated from a WGD in the distant past [23]. This paucity of extant gene duplicates with low synonymous divergence in the *S. cerevisiae *genome led Gao and Innan [27] to conclude an extremely low gene duplication rate of approximately 0.001 to 0.006% per gene per million years for this species. However, a recent study utilizing multiple mutation accumulation lines of *S. cerevisiae *conclusively demonstrates that the spontaneous rate of gene duplication is high, at 1.5 × 10^-6 ^per gene per cell division [43]. This experimental measure in conjunction with the low incidence of extant evolutionarily young gene duplicates in the yeast genome suggests that the fate of most newly spawned gene duplicates in the yeast genome is loss. The large effective population size (*N*_*e*_) achieved in yeast cultures dictates that new gene duplicates with even slightly deleterious selection coefficients may be subject to loss by purifying selection due to the efficacy of natural selection within the yeast genome. The role of effective population size (and, hence, strength of selection) in influencing patterns of genomic sequence evolution has been recently championed by Lynch and colleagues [44-46], although the associated theoretical underpinnings in relation to molecular sequence evolution can be traced back to the proponents of the neutral theory [47,48].

The extant group of gene duplicate pairs with low synonymous divergence in the *S. cerevisiae *genome comprise a mixed population. Most of these pairs (approximately 69%) are derived from evolutionarily older duplications wherein sequence divergence between paralogs has been curbed by the processes of codon selection usage bias, sometimes in conjunction with gene conversion [19,27,28], whereas a smaller subset of gene duplicates (approximately 31%) referred to as non-ohnologs in this study are thought to be of relatively more recent origin, probably occurring subsequent to the WGD event. Furthermore, codon selection usage bias/gene conversion appears to have affected sequence evolution in some of these non-ohnologs as well given that different paralogous pairs within the same linked set (presumably arising from the same duplication event) have extremely divergent K_*S *_values (Table 2). For these reasons, K_*S *_values between gene paralogs cannot be taken as a blanket proxy for estimating the evolutionary age of all gene duplicates, at least in the *S. cerevisiae *genome. The mixed nature of this population of yeast gene duplicates is also apparent during sequence alignments of ribosomal protein paralogs comprising at least one intron. Twenty-four pairs of ribosomal protein yeast duplicates in the ohnolog class have no discernible sequence identity over most of their intronic regions (barring small sequence tracts ranging from 1 to 10 bp at their splice junctions), despite relatively low levels of synonymous divergence in their coding sequences. This lends credence to view that these previously classified ohnologs are indeed of older evolutionary origin [19,23]. Given the presence of ancient gene duplicates with low degrees of synonymous divergence in the *S. cerevisiae *genome, it is reasonable to question whether gene duplicates with low synonymous divergence in other genomes are necessarily young, evolutionarily speaking. A preceding study applied statistical tests for detecting gene conversion to a subset of gene duplicates in the *C. elegans *genome and found that most gene conversion events were restricted to members of large gene families [49], suggesting that the degree of synonymous divergence may be an accurate indicator of evolutionary age for paralogs belonging to small gene families in this genome. Therefore, the worm and yeast genomes may differ in the degree to which concerted evolution or codon usage bias selection effectively homogenizes gene paralogs based on the size of the gene family and the effective population size of the species (and, hence, the strength of natural selection).

We charted out the extent of homology between two paralogs by aligning their genic as well as upstream and downstream flanking regions, thereby calculating a minimal estimate of the extent of duplication by visual inspection. For evolutionarily older duplicates, erosion of sequence homology in the intergenic regions would lead us to underestimate the original duplication span. This expectation is borne out by the fact that 56 of the 93 duplicate pairs in our data set appear to involve the duplication of a single locus. Yet, preceding studies have identified 46 of these 56 gene duplicate pairs as ohnologs. The remaining 37 duplicate genes were generated by 12 duplication events referred to as 'linked sets' (16% of all duplications in this data set) that involved the simultaneous duplication of multiple gene loci (range two to seven genes). Interestingly, only one of these twelve duplication events is thought to have originated from the WGD, suggesting that duplication of lengthier DNA segments encompassing multiple loci is an ongoing process in the yeast genome. Indeed, gene duplication during experimental evolution in yeast frequently involves large chromosomal blocks comprising multiple loci [43,50,51]. Segmental duplications in *C. elegans *encompassing more than one locus, on the other hand, only comprise 7.1% of all observed duplications [34]. This contrast in the patterns of segmental duplication between worm and yeast suggests that duplication events spanning multiple loci occur with a greater frequency and/or are selectively advantageous in the yeast genome relative to *C. elegans*.

Based on a determination of the extent of sequence homology visible between yeast paralogs in their flanking regions, we calculated the minimum duplication span for each duplicate pair and also determined the minimum number of loci that appear to be duplicated. In the majority of the cases, the duplications appear to span only a single locus (approximately 82%; 56 of 68) and the median duplication span for the cumulative data set comprising both ohnologs and non-ohnologs in yeast is 1,004 bp, slightly lower than the median duplication span of 1.4 kb for *C. elegans *gene duplicates. These results appear paradoxical when we consider that the majority of yeast duplicate pairs comprising this data set (69%; 47 of 68) originated via a WGD event. The median duplication span for ohnologs is significantly lower than that for non-ohnologs (958 bp and approximately 2,500 bp, respectively). Furthermore, ohnolog duplication spans are far more restricted in their size range than non-ohnologs. This shorter span of duplication for gene duplicates arising from a WGD are in accord with an older evolutionary age for ohnologs in conjunction with the erosion of sequence homology in their intergenic regions over evolutionary time due to sequence divergence, deletions and/or local rearrangements.

Yeast paralogs were characterized as possessing complete, partial or chimeric structural homology based on the extent of sequence homology using techniques previously described for *C. elegans *paralogs [11]. The genomes of these two eukaryotes are in stark contrast with respect to the frequency of these three structural categories of gene duplicate pairs. The *C. elegans *genome has a high frequency of structurally heterogeneous gene duplicates, with approximately 50% of all evolutionarily young gene duplicate pairs categorized as partials or chimerics [11]. *S. cerevisiae*, on the other hand, has a preponderance of complete duplicates, a handful of chimeric duplicates and a complete absence of partial duplicates. When yeast duplicates are partitioned based on their mechanism of duplication, ohnologs and non-ohnologs are found to be similar with respect to the frequencies of these three structural categories of duplicates. Several factors in combination probably contribute to the paucity of structurally heterogeneous duplicates in the yeast genome. Given a WGD origin for the majority of these duplicates, they are likely to have originated as structural replicas of the ancestral copy with concomitant inheritance of the full repertoire of ancestral *cis*-regulatory elements. Evolutionarily older duplicates such as the ohnologs in this data are likely to have experienced local rearrangements, insertion or deletions that could potentially convert one or both paralogs such that the paralogs appear structurally heterogeneous. However, we observe a remarkable level of structural preservation between evolutionarily older paralogs in *S. cerevisiae*, suggesting purifying selection against mutations modifying ancestral ORF structure and/or pervasive gene conversion leading to structural homogeneity. Indeed, gene conversion is known to operate at an appreciable frequency in the yeast genome and is commonly invoked as one of the factors responsible for the low synonymous divergence among *S. cerevisiae *ohnologs [19,27,28].

Despite the fact that both yeast non-ohnologs and *C. elegans *gene duplicates resulted from SSD events, it is interesting to note that the genomes of these two species differ with respect to the degree of structural homogeneity observed between paralogs. Approximately 82% of yeast non-ohnologs are structurally homogeneous compared to only 40% of gene duplicate pairs with low synonymous divergence in the *C. elegans *genome [11]. This difference may be attributed to an interplay between the median gene length, median duplication span and the strength of natural selection in these two genomes. The median gene length in *S. cerevisiae *and *C. elegans *is 1.1 and 1.4 kb, respectively. The median duplication span for extant *S. cerevisiae *(minimal discernible estimate and excluding ohnologs) and *C. elegans *duplicates is 2.5 and 1.4 kb, respectively. If the median duplication span of extant yeast duplicates accurately approximates that of the entire population of gene duplicates (both preserved and extinct), a SSD event in *S. cerevisiae*, on average, is more likely to encompass the entire ORF of the ancestral copy relative to *C. elegans*. It is also possible that the average length of a SSD event in *S. cerevisiae *may be much shorter than that of extant duplicates. If newly originated duplicates are mildly deleterious because they lack structural and functional redundancy with the progenitor copy, they may be rapidly weeded out in the yeast genome owing to the greater efficacy of natural selection. However, a recent study demonstrates that most spontaneous duplications in yeast experimental lines tend to be fairly large [43]. A smaller *N*_*e *_for *C. elegans *relative to yeast means that such structurally heterogeneous gene duplicates, if mildly deleterious, may be more likely to persist in the worm genome due to an attenuated strength of natural selection.

The genomic location of paralogs relative to one another can provide clues to the mechanism(s) of duplication and the general patterns of their genomic movement subsequent to their origin. Overall, 82% of duplicate pairs in this yeast data set comprise paralogs located on different chromosomes, a pattern that is not surprising given that the vast majority of these gene duplicates are ohnologs that owe their origin to the WGD. Barring the possibility of misidentification of non-ohnologs as ohnologs, the presence of ohnologs with both copies residing on the same chromosome can probably be explained by the secondary movement of one paralog in proximity to its sister copy in the post-duplication period. Interestingly, ohnologs and non-ohnologs display no significant differences with respect to the chromosomal location of paralogs (same versus different chromosomes). While genome- or chromosome-wide duplication events are expected to initially yield paralogs residing on different chromosomes, SSD events do not necessitate such a pattern of paralog location. While approximately 90% of newborn gene duplicates in the *C. elegans *genome comprise both copies residing on the same chromosome [11], only 29% of yeast non-ohnologs are in such close genomic proximity. If gene duplication by retrotransposition is a frequent mechanism of duplication in the yeast genomes due to the presence of *Ty *elements [43,52-55], there should be a further decrease in the likelihood of a paralog originating on the same chromosome as the ancestral locus. However, we have no evidence for the origin of gene duplicates via retrotransposition in this yeast dataset. That is to say, wherever introns are present, both yeast paralogs bear them. Duplications in experimental yeast populations are frequently translocative [43,50]. Furthermore, there is evidence that translocated segmental duplicates in yeast have enhanced stability relative to tandem duplications [56]. Both of these factors likely contribute to the preponderance of yeast non-ohonologs residing on different chromosomes.

Functional diversification between paralogs can be effected by both coding and regulatory sequence divergence. Studies focusing on the absence/presence of a correlation between coding sequence divergence and expression divergence across a breadth of model organisms have yielded contrasting results, reporting the two variables as coupled (for example, [36,57-59]) as well as decoupled [35,60-62]. High levels of gene conversion and/or codon usage bias, which serve to homogenize the coding sequences of paralogs, may restrict the potential for expression and functional divergence between them if coding sequence evolution was the only contributing factor to functional diversification. Given these regimes of pervasive gene conversion and/or codon usage bias in the yeast genome, functional diversification via *cis*-regulatory sequence divergence can greatly facilitate functional diversification of paralogs, independent of coding sequence divergence. Papp and colleagues [63] demonstrated a rapid reduction in the number of shared *cis*-regulatory motifs between yeast duplicates as a function of increasing synonymous divergence despite constancy in the total number of regulatory motifs. Our analysis of the extent of sequence homology in the 5' and 3' flanking regions of yeast paralogs suggests extremely limited levels of sequence preservation in the flanking regions of yeast paralogs, for ohnologs and non-ohnologs alike; 80% and 86% of yeast gene duplicate pairs have detectable sequence homology of only 0 to 10 bp in their 5' and 3' flanking regions, respectively. This diminished sequence identity between paralogs in their flanking regions can be explained by sequence divergence of initially paralogous regions by mutational saturation over evolutionary time, deletions and other rearrangements or a failure to inherit ancestral regulatory elements during the duplication process. Given that many of these gene duplicate pairs are thought to have arisen from a WGD event, the first two scenarios are the most likely explanations for the limited flanking region homology between putative ohnologs comprising this data set. Irrespective of the specific mechanism driving the divergence of flanking regions of *S. cerevisie *paralogs, there exists an appreciable potential for functional diversification between paralogs due to the lack of shared regulatory elements despite complete sequence homology across their ORFs. The causes for the lack of shared flanking region sequence between yeast paralogs are likely to differ for the ohnolog and non-ohnolog classes (rapid molecular divergence versus limited duplication span). However, the sequence divergence in flanking regions of both classes of yeast duplicates is likely to play an important role in driving expression divergence between yeast paralogs, despite the maintenance of sequence homology in their coding regions. Interestingly, ohnologs and non-ohnologs show both similarities and disparities with respect to their flanking region homology. Ohnologs and non-ohnologs were not found to be statistically different with respect to the extent of 5' sequence homology. These results are not in agreement with a previous study that found ohnologs to have more diverged upstream regulatory regions relative to non-ohnologs [25], although this discrepancy between the two studies could be due to both differences in sample size and methodology. In contrast to our 5' flanking region results, there exists a significant difference in the extent of 3' sequence homology between these two classes of yeast duplicates, with ohnologs displaying far more restricted 3' flanking sequence homology relative to non-ohnologs. It is reasonable to suggest that this highly limited extent of homology in the downstream flanking regions of ohnologs is due to diminished selection for conservation of sequence in this area relative to the upstream flanking sequence.

## Conclusions

Ohnologs and non-ohnologs initially need to be considered as separate populations of gene duplicates in the *S. cerevisiae *genome, given their disparate mechanisms of origin as well as their evolutionary ages [24-26]. In general, we find yeast ohnologs and non-ohnologs both share as well as differ in their genomic attributes, with the latter occurring often in unexpected directions. The older evolutionary age of the ohnologs with the concomitant erosion of intergenic sequence homology make them superficially appear as single-locus duplications, akin to other gene duplicates resulting from SSD events. Both ohnologs and non-ohnologs comprise similar frequencies of complete, partial and chimeric duplicates with a strong trend towards a paucity of structurally heterogeneous gene duplicates (partial and chimeric), suggesting a strong role for purifying selection in their elimination. In addition, ohnologs and non-ohnologs do not differ with respect to the chromosomal location of the paralogs, despite presumably disparate mechanisms of duplication leading to their origin. Finally, ohnologs and non-ohnologs both appear to have extremely limited tracts of sequence homology in their upstream and downstream flanking regions, suggesting a possibly greater role for regulatory elements in expression and functional divergence between yeast paralogs. In addition, we concur with other studies that the disparate mechanisms of origin for ohnologs and non-ohnologs may dictate divergent evolutionary fates and trajectories for these two classes of gene paralogs due to varying levels of gene-dosage selection [25,64-66]. However, we conclude that, for the most part, yeast ohnologs and non-ohnologs tend to appear remarkably similar in their structural attributes and genomic locations.

The patterns and features of *S. cerevisiae *gene duplicates show notable differences relative to their counterparts in another model eukaryote, the nematode *C. elegans*. The physical organization and location of the gene duplicates in the two genomes provide evidence for differential mechanisms of duplication. A whole genome duplication event is known to have occurred in the ancestor of *S. cerevisiae*, thereby contributing to different chromosomal locations of the majority of yeast paralogs while these are relatively rare in *C. elegans *[11,31,34]. Additionally, yeast paralogs from SSD events are more likely to be found on different chromosomes as well, either due to translocative duplications, association with mobile elements or selective maintenance [32]. Moreover, the near complete absence of structurally heterogeneous gene duplicates in *S. cerevisiae *also suggests a role for purifying selection in their elimination from the genome. A large *N*_*e *_for *S. cerevisiae *results in greater efficacy of natural selection, which may serve to weed out partial and chimeric duplicates if they are even mildly deleterious with respect to function in their early evolutionary life. We conclude that these differences among gene duplicates in yeast and worm reflect both variable duplication regimes as well as varying strengths of selection owing to the differences in the effective population sizes of the two species.

## Materials and methods

### The *S. cerevisiae *genome

The genome of *S. cerevisiae*, the first eukaryotic genome to be sequenced, is approximately 12 million base pairs (Mb) in length and organized across 16 chromosomes [29]. This genome is relatively compact with almost 70% of the genome comprising ORFs. According to the latest annotated version in the *Saccharomyces *Genome Database [67], the genome encodes 4,649 ORFS, which means that a protein-coding gene is located every 2.6 kb of genome sequence. Of the 4,649 ORFs, 70.34% have been verified experimentally while 17.32% and 12.33% have been assigned to the 'uncharacterized' and 'dubious' categories, respectively.

### Identification of gene duplicates with low synonymous divergence in the *S. cerevisiae *genome

The complete set of available nucleotide sequences for all putative ORFs in the *S. cerevisiae *genome were downloaded from the *Saccharomyces *Genome Database [67]. A WU-BLAST was used to query each ORF nucleotide sequence against all other sequences in this data set, retaining those pairs with E-values less than 10^-6 ^to reduce the frequency of chance alignments. The resulting BLAST reports were further screened to identify alignments with at least 90% sequence identity and lengths exceeding 30 bp. We excluded multigene families involving three or more members, focusing entirely on cases with only two gene copies in the *S. cerevisiae *genome.

### Identification of duplication termination points and linked groups

Using the final set of BLAST alignments as a guide, we proceeded to retrieve the ORF nucleotide sequence (both the spliced and unspliced if intron(s) were present) as well as 5 kb of upstream and downstream nucleotide sequence for both putative paralogs. All nucleotide sequences corresponding to the two paralogs were visualized and aligned in Se-AL, version 2.0A11 [68]. We aligned the 5' upstream and 3' downstream nucleotide sequences of the paralogs, accessing additional sequence if necessary, until no homology was apparent for 2 kb. This enabled us to identify the duplication termination points, calculate the length of the duplication span [11] and determine if additional ORFs aside from the focal loci comprising the duplicate pair were included in the duplication event.

### Calculation of synonymous and nonsynonymous substitutions

For each set of paralogs, we calculated the number of nucleotide substitutions per synonymous and nonsynonymous site using the Nei and Gojobori method [69] corrected for multiple hits via the online molecular software SNAP [70].

### Characterization of duplicate pairs into structural categories

We also used the alignments of spliced, unspliced (if available) and upstream/downstream flanking regions of the two paralogs to determine the degree of structural resemblance between them, as per the protocol used in Katju and Lynch [11]. Gene duplicates with complete structural resemblance exhibit complete sequence homology between the initiation and termination codons. For gene duplicates with amino acid sequences of differing lengths, duplicates were still designated as possessing complete structure if the shorter copy exhibited nucleotide sequence homology to the lengthier copy throughout the latter's ORF, irrespective of differential annotation, if present, with respect to exon-intron and flanking region boundaries. Insertions/deletions (indels) resulting in frameshifts or in-frame gaps were ignored as long as nucleotide sequence homology between the two copies was resumed within the ORF boundaries of the lengthier reference sequence, before the start of flanking region(s). Partial duplicates comprised paralogs of differing amino acid lengths wherein the entire ORF of the shorter gene was homologous to the lengthier gene's ORF but the latter ORF had unique sequence to the exclusion of the shorter gene copy. Finally, chimeric duplicates comprised cases wherein both paralogs, in addition to homologous regions, had unique ORF sequence to the exclusion of the other gene copy.

### Determination of duplication span

The extent of minimal duplication span for each duplicate pair was measured by initially aligning the ORF regions as well as 2 kb of 5' and 3' flanking regions for each paralog against the other. Duplication termination points in both the 5' and 3' directions were identified as the nucleotide beyond which no homology was apparent between the paralogs for a continuous stretch of 1 kb on either end despite accounting for indels. The duplication span was measured as the length of sequence between the 5' and 3' duplication termination points. This methodology therefore underestimates the true duplication span at the time of the duplication event and only offers a minimal estimate of the extent of homology still apparent between the two paralogs. For example, in the instance of an indel exceeding 1 kb in one paralog, we would fail to detect the resumption of homology between the two copies beyond the indel location. Likewise, we would prematurely designate duplication termination points in intergenic sequence tracts wherein sequence homology between the paralogs has been eroded due to rapid rates of molecular evolution or a lack of selective pressure for sequence conservation.

## Abbreviations

Indel: insertion/deletion; *N*_*e*_: effective population size; ORF: open reading frame; SSD: small-scale duplication; WGD: whole-genome duplication.

## Authors' contributions

VK and UB designed the experiment; VK and JCF performed the research; VK and UB wrote the paper.
